# Long-Term Central and Effector SHIV-Specific Memory T Cell Responses Elicited after a Single Immunization with a Novel Lentivector DNA Vaccine

**DOI:** 10.1371/journal.pone.0110883

**Published:** 2014-10-22

**Authors:** Géraldine Arrode-Brusés, Maha Moussa, Monique Baccard-Longere, François Villinger, Yahia Chebloune

**Affiliations:** 1 INRA, ANRS, Université Joseph Fourier, PAVAL Lab./Nanobio 2, UJF Grenoble, Grenoble, France; 2 Institut de Biologie et Pathologie, Centre Hospitalo-Universitaire de Grenoble, Grenoble, France; 3 Division of Pathology, Yerkes National Primate Research Center, Emory University, Atlanta, Georgia, United States of America; Centers for Disease Control and Prevention, United States of America

## Abstract

Prevention of HIV acquisition and replication requires long lasting and effective immunity. Given the state of HIV vaccine development, innovative vectors and immunization strategies are urgently needed to generate safe and efficacious HIV vaccines. Here, we developed a novel lentivirus-based DNA vector that does not integrate in the host genome and undergoes a single-cycle of replication. Viral proteins are constitutively expressed under the control of Tat-independent LTR promoter from goat lentivirus. We immunized six macaques once only with CAL-SHIV-IN^−^ DNA using combined intramuscular and intradermal injections plus electroporation. Antigen-specific T cell responses were monitored for 47 weeks post-immunization (PI). PBMCs were assessed directly *ex vivo* or after 6 and 12 days of *in vitro* culture using antigenic and/or homeostatic proliferation. IFN-γ ELISPOT was used to measure immediate cytokine secretion from antigen specific effector cells and from memory precursors with high proliferative capacity (PHPC). The memory phenotype and functions (proliferation, cytokine expression, lytic content) of specific T cells were tested using multiparametric FACS-based assays. All immunized macaques developed lasting peripheral CD8^+^ and CD4^+^ T cell responses mainly against Gag and Nef antigens. During the primary expansion phase, immediate effector cells as well as increasing numbers of proliferating cells with limited effector functions were detected which expressed markers of effector (EM) and central (CM) memory phenotypes. These responses contracted but then reemerged later in absence of antigen boost. Strong PHPC responses comprising vaccine-specific CM and EM T cells that readily expanded and acquired immediate effector functions were detected at 40/47 weeks PI. Altogether, our study demonstrated that a single immunization with a replication-limited DNA vaccine elicited persistent vaccine-specific CM and EM CD8^+^ and CD4^+^ T cells with immediate and readily inducible effector functions, in the absence of ongoing antigen expression.

## Introduction

More than three decades after the discovery of HIV, the development of a safe and efficacious vaccine that can induce protective immunity in humans against HIV/AIDS remains an unfulfilled priority. The classical vectors and strategies for vaccine development, efficient for acute infectious diseases, have failed to prevent acquisition and/or control of acquired HIV-1 infection. These results indicate that novel vectors/strategies need to be explored and developed to induce protective immunity against this type of persistent infection. One significant hurdle to this progress is the fact that correlates of protection are not fully elucidated [1]. Among naturally infected HIV-1 patients, few individuals such as Long-Term Non-progressors (LTNP), Elite suppressors (ES) and recently the Berlin patient have shown successful control of replication of their lentiviral infection [2]–[4]. However, in some of these patients, HIV-1 variants naturally attenuated by mutation in the *nef* gene (Live-attenuated) were isolated [5]–[8]. This observation provided a rationale for testing live-attenuated (LAV) SIV and SHIV vaccines in non-human primate (NHP) models. LAV especially those with the least attenuated design, remain the only “vaccines” found to be able to achieve reproducible protection in macaques challenged with highly pathogenic viruses [9]–[12]. One salient safety issue associated with these “vaccines”, is the fact that they cause a persistent infection associated with integration of the provirus into the genome of the host, leading to potential mutations and gain of virulence especially in infants and in some adult macaques [13]–[16]. Nevertheless, the protective responses afforded by LAV warrant additional investigation into mechanisms of protection [17] and similar approaches with hopefully better safety profiles, i.e. viral vectors that will mimic natural exposure to the virus but without integration into the genome and self-limited replication. Thus, genetic systems were developed to produce strains of SIV whose replications were limited to a single-cycle, leading to the production of virus proteins or virus like particles (VLPs). In particular, macaques repeatedly immunized with single-cycle SIV particles mounted potent virus specific T cell responses which did not prevent infection but significantly contained SIV replication after challenge [18], [19], but to a lesser extent than persisting live-attenuated vaccine [17]. These results suggested that the ongoing stimulation of virus-specific immune responses might be essential to achieve long-term protection. The correlates of protection upon continuous antigen expression for the maintenance of vaccine-specific T cells associated with immediate antiviral effector functions have recently been highlighted in LAV-mediated protection in an NHP study [17]. In addition, persistent and replication-competent recombinant viruses, such as cytomegalovirus vector expressing SIV antigens, provided complete protection in a subset of vaccinated monkeys, demonstrating that the continuous presence of vaccine-specific effector memory (EM) T cells can lead to complete control of SIV [20], [21]. Interestingly, in a mouse model of chronic infection with lymphocytic choriomeningitis virus (LCMV), a cooperative maintenance of progenitor and terminal progeny of virus-specific CD8^+^ T was found to be essential to maintain a durable and effective control of viral replication during chronic infection [22]. Altogether, these results have supported the idea that protective responses may require a combination of T cell responses with characteristics of both memory and effectors cells.

We and others [23]–[25] have focused on the use of DNA alone as a vaccination strategy because DNA is a simple, safe and stable component, obviating concerns about preexisting immunity and suitable for the development of large scale vaccination. Our group’s strategy focuses on the development of a HIV DNA vaccine based on modified lentiviral genomes to better mimic natural exposure to HIV and production of viral proteins. In earlier studies, deletions of *vpu,* and *nef* were tested in the context of the pathogenic SHIV-_KU2_ infectious molecular clone to develop LAV vaccines. Inoculated NHP were protected from challenge with highly pathogenic viruses [12] but this protection had a finite duration [26]. Additional truncation of *rt, integrase* and *vif* genes along with the 3′ LTR from SHIV-_KU2_ genome yielded Δ4-SHIV-_KU2_, a non-replicative and non-integrative DNA vaccine which allowed the assembly of VLPs that served as potent antigen to prime T cells responses. NHP vaccinated repeatedly with our DNA vaccine only, were protected from the challenge with SHIV89.6p [27], [28], but this DNA immunization induced no detectable humoral responses and only modest levels of cell-mediated responses [29]. Improved responses were achieved using a single administration of a high dose of Δ4-SHIV-_KU2 _DNA in mice and NHPs [30], [31]. These studies demonstrated life-long SHIV-specific T cells responses against Gag and other HIV antigens in the animal models. These cells displayed polyfunctional properties (proliferation, lytic content but limited effector functions) upon *in vitro* restimulation. In macaques also, persistent low levels of long lasting vaccine-specific T cells were induced, without antigen boost *in vivo*, which were expanded further *via* the administration of IL-15 *in vivo* [31].

We hypothesized that the modest immune responses may be secondary to limited production of antigen and recently substituted the tat responsive SIV LTR with the LTR from a naturally attenuated lentivirus, caprine arthrithis encephalitis virus (CAEV), which promotes the expression of proteins constitutively [32], [33]. Of importance, this LTR substitution maintained the immunogenicity of the SHIV-_KU2_-based vaccine after a single dose in mice and macaques [34]. For the current study, the SIV *integrase* (*IN*) gene was deleted from the SHIV-_KU2_ genome and the SIV LTR were substituted with those from CAEV to generate CAL-SHIV-IN*^−^*. This novel DNA vaccine, is designed to be administered as plasmid DNA leading to the production of viral antigens that will be assembled into VLPs as well as pseudo-infectious particles that have the potential to cause one cycle of infection in target cells without integration of the viral genome. Since our intramuscular (IM) only Δ4-SHIV-_KU2 _DNA vaccine studies induced only few systemic T cells with immediate effector functions, we also applied intradermal (ID) injections plus electroporation (EP) of the CAL-SHIV-IN*^−^* DNA vaccine in addition to the classical IM injection. In this report, we performed a longitudinal phenotypic and functional analysis of vaccine-induced T cell responses in a NHP model across a year post-immunization.

## Materials and Methods

### SIV and HIV peptides

Overlapping 15-mer peptides, with 11-aa overlaps, spanning the entire SIV Gag, Pol and Nef and HIV Env, Tat, and Rev proteins were obtained from the National Institutes of Health AIDS Research and Reference Reagent Program (catalog nos. 6204, 8762, 6443, 6451, 5138 and 6445, respectively). HIV Env, Tat and Rev peptides are based on the consensus sequences from clade B HIV genomes. Our SHIV-based DNA vaccine encodes Env from the HXB2 and Tat and Rev from the SF2 HIV strains. These two strains are both clade B viruses. The *gag*, *nef* and *pol* genes were derived from the SIVmac239 genome. Individual SIV Gag GW9 (GPRKPIKCW) and SIV Nef RM9 (RPKVPLRTM) peptides were kindly provided by Dr. Roger Le Grand, (CEA, Fontenay-aux-Roses, France).

### Animals

Six 3–5 year-old male cynomolgus macaques were purchased from Bioprim, SA (Toulouse, France), imported from Mauritius and were housed in the Cynbiose facility, Lyon, France. At reception, complete examination under ketamine sedation (IV injection, 10 mg/kg), was conducted by a veterinarian, and included abdominal palpation and observations of the condition of integument, respiratory and cardiovascular systems.

Animals were housed in enclosures in groups of three, except during treatment and clinical examination. They were in temperature controlled rooms with monitored air conditioning system (>10 air changes per hour, 22°C +/− 3°C, 12/12 hours of light/dark photoperiod). As macaques can spend many minutes at a time manipulating their environment, for environmental enrichment toys were included in each individual cage. In addition each room for housing the macaques was equipped with mirrors facing the cages and intermittent diffusion of selected music. Cleaning of animal rooms and cages were performed on a daily basis.

Feeding of animals was with specific primate diet (SDS OWM ref: 818004 and Sniff ref: D-59494) provided daily in appropriate (100 g for animals under 5 kg and 200 g for animals over 5 kg). Delicacies were also occasionally given to the animals as part of the Testing Facility environmental enrichment program.

For drinking Tap water was available *ad libitum* to each animal via an automatic watering device.

The study animals were acclimated to their designated housing for at least 28 days prior to the first study day. None of these animals was sacrificed or died during the study. All the animals are still alive and kept for further studies.

### Statistical analysis

Primarily, we examined the number of animals that will develop immune responses specific to SHIV antigens in vaccinated macaques. We used Fisher’s Exact test to evaluate the number of animals that develop immune responses assuming a one-sided type I error rate of 5%, all six vaccinated animals gives us 70% power to detect enhanced immune responses to 68% and 80% power to detect development of specific immune responses to 85% in the vaccinated group. Thus, we had sufficient reason to conclude that vaccinated animals with SHIV-based DNA vaccine were developing strong and specific immunity against SHIV if 5 or more of the 6 vaccinated animals develop potent immunity specific to SHIV antigens compared to the initial findings that none of these animals had any specific immune response against SHIV antigens.

### Ethics Statement

All macaques were used in accordance with the guidelines of the EU Directive 86/609/EEC as published in the French Official Journal of February 13^th^ 2001 for experiments using non-human primates. The study plan was reviewed by the Regional Ethics Committee of VetAgroSup (1, Avenue Bourgelat, 69280, Marcy l’Etoile, France) and approved under permit protocol number 1105. Cynbiose SA is accredited by the Direction of Veterinary Services, (DVS #691270505). Immunizations and the collection of blood samples at each time point were performed under ketamine tranquilization (10 mg/kg) to minimize pain and suffering.

### MHC haplotype analysis

Genomic DNA was extracted from peripheral blood using the QIA amp Blood Kit (Qiagen, Courtaboeuf, France) as recommended by the manufacturer. Genotyping of Major Histocompatibility Complex (MHC) was performed using 20 microsatellites markers scattered across the MHC region as described previously [35], [36]. This type of analysis identified seven common haplotype from H1 to H7 in Mauritian cynomolgus macaques.

### Vaccine CAL-SHIV-IN^−^ plasmid DNA

CAL-SHIV-IN*^−^* is a derivative of the pathogenic SHIV-KU2 from which the 5′ and 3′ SIV LTRs were exchanged with the constitutively active CAEV LTRs. In addition, the SIV *integrase* coding sequences were removed (Figure S1). Thus, the CAL-SHIV-IN*^−^* expresses SIV Gag, Pol (minus *integrase*) Vif, Vpx, Vpr and Nef as well as HIV Vpu, Tat, Rev and Env proteins.

### Immunization of macaques

Macaques were immunized with a total 5 mg of DNA vaccine injected only once in multiple sites and 2 different delivery routes. The DNA solution was prepared in sterile PBS at 1 mg/ml. All DNA used contained at least 90% of supercoiled plasmid. Animal were put to sleep following injection of 10 mg/kg of ketamine. Intramuscular inoculations (2 mg/site) were delivered to each rear leg using a 19 gauge needle. Intradermal injections were spread over 10 different sites (0.1 mg/site) under the skin of the back, after shaving. In addition, the ID immunizations were followed by electroporation comprising of six successive 10-msec square-wave pulses, output current 300–600 mA, with 90-msec intervals between pulses as previously described [37]. A portable pulse generator (CUY21 EDIT; Nepa gene, Ichikawa, Chiba, Japan) and Tweezer electrodes were used, with a conductive gel to ensure good contact between the electrodes and skin. This electroporation device was gently provided by Dr. Le Grand (CEA, Fontenay-aux-Roses, France) for the duration of the immunization experiment.

### Detection of anti Env gp160 and anti-Gag p55 antibodies

For anti-Env gp160 antibody detection we used a commercial ELISA kit, the Genscreen kit ULTRA HIV Ag-Ab (Bio-Rad, France). Serum samples from the macaques were diluted 1/8 in 1xPBS and loaded into the 96-well plates pre-coated with HIV Env gp160 antigen. The ELISA was performed by an automatic machine following the protocol provided by the manufacturer (BEP III System, Siemens).

For anti-SIV Gag antibody detection we developed and standardized a “home-made” ELISA using a recombinant SIVmac Gag p55 antigen. Microtiter 96-well plates (Nunc, France) were coated with 8 ng/well in 100 µl of SIV Gag p55 diluted in 0.1 M sodium bicarbonate (pH 9.6). After overnight incubation at 4°C, the plates were washed thrice with 200 µl/well with 1xPBS-0.1% Tween (PBS-T). The plates were then saturated with 1% BSA, (100 µl/well) following 2 hours incubation at room temperature and then washed with PBS-T. Serum samples from macaques were diluted 1/8 in PBS-T and loaded in triplicates. The plates were incubated 1 hour at 37°C and then washed thrice with PBS-T. A secondary antibody (polyclonal goat anti-human IgG conjugated with horseradish peroxidase) was used at dilution 1/15,000 (100 µl/ml) and incubated 1 hour at room temperature. Plates were then washed thrice with PBS-T prior to adding the substrate, 100 µl/well of tetra-methyl-benzidine (TMB) and incubation 5 minutes at room temperature. The reaction was stopped by adding 100 µl/well of stop solution (1N H_2_SO_4_). The plates were read in an ELISA reader at 450 nm.

### Detection of IFN-γ-producing cells by ELISPOT assay

We used a commercial non-human primate ELISPOT kit (Mabtech, France) to evaluate IFN-γ-producing cells in response to pools of overlapping peptides. Briefly, 2.5×10^5^ peripheral blood mononuclear cells (PBMCs) per well were incubated *in vitro* for 18 h with complete pools of peptides for Gag, Env, Tat+Rev+Nef (TRN) and Pol (1 µg of each peptide pool/ml), medium alone (used as negative control), medium containing anti-CD3 mAb (used as positive control) or individual Gag and Nef peptide (5 µM/ml) when indicated. Spots were counted using an automated ELISPOT reader system (Bioreader 500-Eb, Biosys). Results are presented as mean number of IFN-γ spot forming cells (SFC) per million PBMCs and were calculated from triplicate wells after subtracting the mean number from triplicate control wells (medium without peptide). Samples were scored as positive when the net adjusted counts exceeded 50 SFCs per million PBMCs. Alternatively, we performed an assay referred to as the PHPC assay (memory Precursor with High Proliferative Capacity [38]), with slight modifications of the protocol. Briefly, PBMCs were cultured for 11 days at 37°C in 2 ml of serum-free medium (AIM-V) with or without peptides in 24-well tissue culture plates at a density of 2×10^6^ cells/well. On day 3, cultures were supplemented with simian IL-2 (10 U/ml) only and on day 7 with a cocktail containing simian IL-2, IL-15 (10 U/ml each, from the Resource for NHP immune Reagents) and IL-7 (500 ng/ml, gift from Cytheris, Yssy-les-Moulineaux, France). On day 11, expanded cells were counted and 5×10^4^ PBMCs per well were used for IFN-γ-ELISPOT assay and the remaining cells for polychromatic flow cytometry analysis.

### Flow cytometry assays for SHIV-specific immune T cells

Polychromatic flow cytometry analysis was performed using a three-laser BD LSRII instrument and data files were collected and analyzed using the FACSDiva software (version 6.1.3; BD Biosciences). We assessed the memory phenotypes and effector functions of SHIV-specific T cells on PBMCs freshly isolated or cultured for 11 days as described in the PHPC assay. The proliferation and effector functions were also measured in CFSE-labeled PBMCs, cultured for 5 days at 37°C in 1 ml of serum-free medium with or without peptides in 96-deep well tissue culture plates at a density of 2×10^6^ cells/well. Fresh or cultured cells were restimulated for 16 h with medium with or without relevant SHIV peptides in the presence of 0.5 µg/ml of costimulatory CD28 (clone CD28.2), CD49 (clone 9F10) mAbs (ebiosciences, France) (except for CM/EM phenotyping) and brefeldin A (Sigma, France). Cells were then washed and stained with different combinations of Abs including Pacific Blue-conjugated anti-CD3 (clone SP34-2), APC-H7-anti-CD8 (clone SK1), PE-anti-CD4 (clone L200), Alexa-700-anti-CD4 (clone L200), PerCPCy5.5-CD28 (clone CD28.2) and PE-CD95 (clone DX2) for 20 min at 4°C. Additionally, ethidium monoazide (EMA; Molecular Probes) was used to allow the exclusion of dead cells. Cells were fixed/permeabilized and stained with different combinations of mAbs including PE-Cy7-anti-IFN-γ (clone B27), alexa-700 anti-Granzyme B (clone GB11), APC-IL-2 (clone MQ1-17H12) and Alexa-488-TNF-α (clone Mab11) mAbs. All mAbs were purchased from BD Biosciences unless otherwise stated. For each experiment, unstained and single-color controls were included to allow proper compensation as well as all-fluorescence-minus-one control to determine proper population gates. Each analysis was gated on low forward and side scatter lymphocytes (FSC/SSC), EMA-, CD3*^+^*, high CD8*^+^* or CD4*^+^* population to collect 25,000–150,000 CD8*^+^* or CD4^+^ events (>10^6^ total events) depending on the assay. Data were displayed as two-color or density dot plots to measure the proportion of single- and double-positive cells in the gated CD3*^+^*CD8*^+^* population (orange color) or CD3*^+^*CD4*^+^* population (blue color). Bioexponential display was also used to show each population in its entirety. For CFSE proliferative responses, pre-immunization samples were used to determine the cut-off for positive responses against each individual mix of peptides in each animal. Proliferative responses above 0.3% of the total CD8^+^ or CD4^+^ T cell populations were scored as positive. In addition, control culture medium was obtained at each time point and condition and background values obtained with cells cultured for 5 days with medium only and restimulated for 16 h with relevant pools of peptides were subtracted.

## Results

### 1. Longitudinal evaluation and characterization of IFN-γ immediate effector cell responses following a single DNA immunization in macaques

#### IFN-γ ELISPOT responses

**Figure 1:**
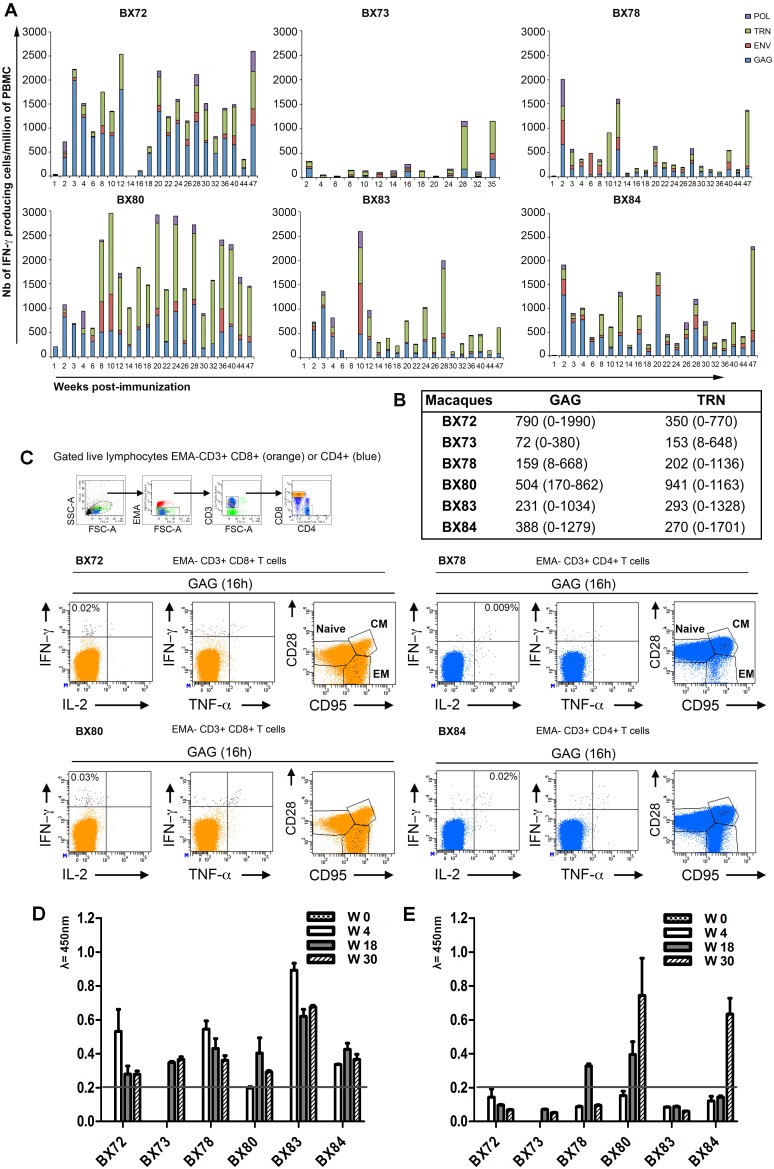
Evaluation and characterization of immediate effector T cell responses induced by a single immunization with the CAL-SHIV-IN^−^ DNA vaccine. **A)** PBMCs isolated from blood samples taken at the indicated weeks post-immunization (PI) were used for ELISPOT assay to detect IFN-γ producing cells in response to the pools of SIV Gag (indicated in blue), HIV Env (indicated in red), HIV Tat+Rev+ SIV Nef (TRN, indicated in green) and SIV Pol (indicated in purple) peptides. Numbers of IFN-γ producing cells obtained per million of PBMCs against all Ags tested are indicated in the *y*-axis. **B)** Mean and range of IFN-γ ELISPOT responses to Gag and TRN pools across 22 (for BX72, 78, 80, 83, 84) and 14 (for BX73) time-points collected. **C)** Memory phenotyping of Gag-specific T cells at W8 PI. PBMCs were restimulated for 16 h in presence or absence of Gag pool of peptides in medium containing Brefeldin A. Cells were then surface stained with EMA, CD3, CD8, CD4, CD28, CD95 mAbs, permeabilized and stained with IFN-γ, IL-2 and TNF-α mAbs. For analysis, cells were initially gated on live lymphocytes (low FSC/SSC, EMA-, CD3^+^), CD8 bright T cells (orange color) and CD4^+^ T cell populations (blue color). Antigen-specific T cells were identified by their capacity to secrete one or more cytokines (black dots). For each indicated animal (BX72, 78, 80, 84), we displayed the maximal response obtained at this time point within CD8^+^ or CD4^+^ T cell populations. For simplicity, the percentage of total responses (total black dots) obtained through all quadrants (IFN-γ+, IFN-γ+ &TNF-α+, IFN-γ+&IL-2+ and IL-2+) are indicated after the subtraction of background obtained with cells cultured with medium only. For memory T cell subset determinations, all black dots were superimposed to the total CD8^+^ or CD4^+^ T cell populations and plotted against memory markers (CD28 and CD95). The naïve population was defined as CD28+ CD95−, effector memory (EM) as CD28− CD95+ and central memory (CM) as CD28+ CD95+. D) Sequential evaluation of SIV Gag p55 specific antibody responses in immunized macaques (“homemade kit”). E) Sequential evaluation of HIV Env gp160 specific antibody responses in immunized macaques (Biorad, France).

We immunized six cynomolgus macaques (BX72, 73, 78, 80, 83, 84) with each animal delivered a single immunization with the CAL-SHIV-IN^−^ DNA vaccine consisting of combined IM and ID-EP (intradermal plus electroporation) administrations as described in material and methods. The vaccine-induced IFN-γ responses were measured longitudinally against Gag-, Pol-, Env- and Tat+Rev+Nef in PBMCs isolated from fresh blood samples collected across a year post-immunization (PI). Samples were analyzed weekly during the first 4 weeks, every other week up to W32 PI and then every 4 weeks up to W47 PI. Monkey BX73 was vaccinated 3 months after the other animals and sampled every other week from W2 to W20 PI and then every 4 weeks up to W35 PI. Data from this longitudinal examination reported in Figure 1A showed that all six animals immunized once with a total of 5 mg of DNA vaccine developed circulating antigen-specific IFN-γ producing T cell responses that persisted until the end of follow-up (W47 PI). While individual variations were noted, most primary expansion for total antigen responses peaked during the first three months (W2 to 12) PI. Interestingly, secondary peaks of expansion occurred, in the absence of any antigen boost, at multiple late time points PI. In five of six animals, maximal peak responses observed during the primary expansion phase or at reemergence time points ranged between 2000 to 3000 spots/million of PBMCs (10^6^). The sixth animal, BX73 mounted lower peak responses at about 1000 spots/10^6^. The magnitude and timing of the responses varied between macaques, however at the level of individual antigens, responses against Gag and TRN were detected in all animals at most time points with mean responses and ranges shown in Figure 1B. For Env and Pol antigens, the calculated overall average responses reached in all 6 vaccinated animals were generally lower (85 spots/10^6^ for Env and 51 spots/10^6^ for Pol), commensurate with the relative lower levels of production of these proteins. Thus, all vaccinated macaques developed broad IFN-*γ* responses directed against all administered antigens with dominant responses against Gag and TRN antigens.

Altogether, these results demonstrated that the IM and ID-EP deliveries of a single immunization with a one cycle SHIV-based DNA vaccine induced long-lasting and broad IFN-*γ* immediate effector cell responses in the peripheral blood of all vaccinated macaques, in absence of boost immunization.

#### SHIV specific antibody responses

Antibody titers against SIV Gag p55 (“homemade”) and HIV Env gp160 (BioRad, France) were also evaluated at various time points post immunization (Figure 1D and E respectively.) As seen in Figure 1D, all animals developed clearly detectable antibody titers to SIV Gag which peaked at week 4, with stable low levels thereafter (Figure 1D). In contrast, only 3 monkeys (BX 80, 84 and 78) developed detectable responses to HIV Env, which unlike the Gag responses tended to increase over time. Overall though these responses remained modest, likely owing to limited production of antigen for B cells.

#### MHC haplotypes

Due to the heterogeneity of the responses observed between macaques, we determined the MHC haplotypes of our Mauritian Cynomolgus macaque (MCM) cohort by performing microsatellite analysis (Figure S2A). Six common haplotypes from H1 to H6 were found but none of the animals were found to be homozygous. Two animals (BX83 and 84) were simple recombinants and four (BX72, 73, 78 and 80) exhibited one or more recombinations. None of them matched the entire MHC class I and class II haplotypes but some (BX72 and 83) shared common MHC class IA and B haplotype and other (BX80 and 83) MHC class II haplotype. One animal, BX78 carried MHC class IB H6 haplotype that is associated with resistance to SHIV89.6P and SIVmac251 [39], [40]. Overall, this type of analysis documented the genetic diversity among our unselected cohort which may have contributed to the variability of the responses. In a previous study, O’Connor and co-workers have shown that two MHC-IA alleles were expressed in more than 88% of MCM suggesting that CD8^+^ T cells responses restricted by these MHC-IA alleles would be detected in nearly all MCM [41]. At the latest time point (W47 PI), we tested the two epitopes associated with these two alleles, commonly named GW9 for Gag and RM9 for Nef antigens. As shown in Figure S2B, all 6 macaques mounted positive IFN-γ responses against Gag GW9 and Nef RM9 antigens in a conventional (18 h) and an extended IFN-γ ELISPOT assay. Due to limited numbers of cells per sample and limited resources in peptides, we could not perform epitope mapping experiments and determine the breadth of vaccine-induced responses. However, these results indicated that our single-cycle vaccine promoted, in all six animals, the development of responses that are generally present only during the early phase of fully replicative SIVmac239 infection and rapidly lost by mutation escape, probably due to the antiviral pressure they exert [41]. Their persistence in our vaccinated macaques suggested an acute antigen expression by our single-cycle vaccine.

#### Polychromatic flow cytometry assay

To further identify the cells involved in these IFN-γ responses, we also performed phenotypic and functional analysis using a polychromatic flow cytometry assay. Gag-stimulated cells for 16 h and medium only control cells were surface stained with anti-CD3, CD4, CD8, CD28 and CD95 mAbs in the presence of EMA (for exclusion of dead cells), permeabilized and then intracellularly stained with anti-IFN-γ, TNF-α and IL-2 mAbs. Analyses performed at W8 PI (Figure 1C and Figure S3) and after W40 PI in four animals, identified Gag specific-CD8^+^ T cells capable of secreting IFN-γ and/or TNF-α as well as Gag-specific CD4^+^ T cells capable of secreting IFN-γ and/or IL-2, suggesting the induction of polyfunctional antigen-specific T cells by the vaccine. At these early and late time points PI, the proportion of single or multiple cytokine secreting cells, circulating in the peripheral blood, was below 0.05% of total live (EMA-, CD3^+^) CD8^+^ or CD4^+^ T cells (median = 0.015%). The vast majority of the SHIV-specific T cells expressed an effector memory phenotype (CD28−, CD95+; EM) for CD8^+^ T cells and central memory phenotype (CD28+, CD95+; CM) for CD4^+^ T cells.

### 2. Polyfunctional analysis of SHIV-specific CD8^+^ T cell recall responses

We previously developed an immune-monitoring assay that measures polyfunctional recall T cell responses and have shown that macaques and mice immunized with a single high dose of Δ4-SHIV-_KU2 _DNA vaccine mounted antigen-specific T cell responses capable of proliferation in response to Ag stimulation for 6 days with limited immediate effector functions but containing lytic granzyme B. These responses developed in the first few weeks post-immunization, then contracted to minimal levels but reemerged later, in absence of any antigen boost, and persisted for at least 63 weeks PI in mice and at least 45 weeks in macaques [30], [31].

**Figure 2:**
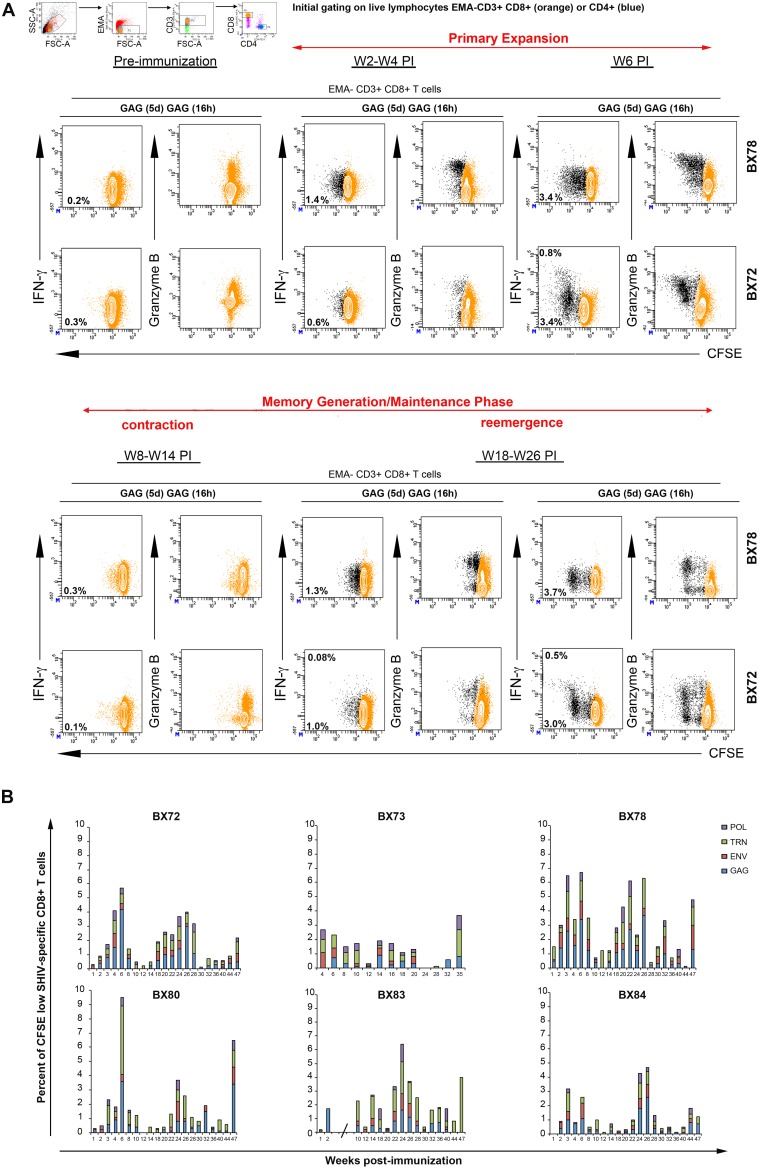
Polyfunctional SHIV-specific CD8^+^ T cell recall responses. At various indicated time points pre- and post-immunization PBMCs from vaccinated animals were labeled with CFSE and cultured in presence of specific pools of SHIV peptides Gag, Env, TRN, Pol or medium without peptide for 5 days. On day 5, cells were harvested and restimulated overnight with the same cocktail of peptides (designated as Gag(5d) Gag(16 h)) in the presence of costimulatory Abs and Brefeldin A. Cells were then surface-stained with EMA, CD3, CD8, CD4 mAbs, permeabilized and stained with IFN-γ, IL-2 and Granzyme B mAbs. For flow cytometry analysis, we initially gated on live lymphocytes (low FSC/SSC, EMA-, CD3^+^), bright CD8^+^ T cell populations (colored in orange). Antigen-specific T cells were identified by their capacity to proliferate, secrete cytokines and contain lytic molecules (black dots). **A)** Results for two representative animals (BX72 and BX78) were displayed. The proportion of cells producing IFN-γ (contour plot; upper number) and proliferating (CFSE dilution, contour plot, lower number) in response to specific antigens is indicated in each plot. Frequencies for antigen-specific responses are reported as the percent of cytokine-secreting and proliferating CD8^+^ T cells after the subtraction of backgrounds obtained with cells cultured for 5 days with medium only and restimulated for 6 h with relevant peptide pools. **B)** Summary of the frequencies of proliferating (CFSE low) CD8*^+^* T cells detected against each indicated antigen (Gag (blue), Env (red), TRN (green), Pol (purple)) in each immunized animal (BX72, 73, 78, 80, 83, 84) at various weeks post-immunization. Of note, results obtained between W3 and W8 for BX83 were excluded due to non-specific T cell hyperactivation (indicated by an interrupted x-axis).

We used the same assay to longitudinally examine the T cell responses from PBMCs in the macaques immunized in the current study. Results from two representative macaques (BX72 and 78) are shown in Figure 2A. During the first 4 weeks PI, Gag-specific CD8^+^ T cells that were detected have moderate capacity of proliferation (<3 divisions), no immediate effector functions (IFN-γ-, IL-2-) but contained the lytic Granzyme B (>80% positive cells). By week 6 PI, cells with higher proliferative capacity (>3 divisions) together with cells producing IFN-γ have developed. However, the proportions of IFN-γ producing cells always represented minor fractions of antigen-specific proliferative T cells and they never exceeded 1% of total CD8^+^ T cell populations at any of the time points or animals tested. After this primary peak, Gag-specific proliferative responses decreased and contracted to levels below detection for several weeks until they started to re-emerge (Figure 2A). These secondary T cell responses appeared at later time points, in the absence of booster immunization, and their profile showed similar properties to those found in the primary peak. These Gag specific CD8^+^ T responses were composed of cells with low and high proliferative capacity with limited immediate IFN-γ responses but containing Granzyme B in more than 50% of the responding cells.

The results of the 47 weeks (35 weeks for BX73) follow-up of proliferative CD8^+^ T cell responses for each antigen tested (Gag, Env, Pol and TRN) and for each animal are represented in the histograms of Figure 2B and summarized in Table S1. This longitudinal study showed that all six animals, receiving a single immunization of 5 mg of DNA vaccine, developed proliferative CD8^+^ T cell responses in their peripheral blood, which persisted for the duration of follow-up. These responses comprised primarily cells with limited immediate effector function (IFN-γ) but with detectable Granzyme B. Maximal responses against all antigens tested, observed during the primary expansion phase or at re-emergence time points, ranged from 2.8 to 9.5% of total CD8^+^ T cell population. The level of these maximal responses varied across time in each animal but remained close to the levels found at the primary peak phase. Regarding individual antigens, each animal mainly exhibited Gag and TRN responses at most time points tested. Among all animals, maximal Gag proliferative CD8^+^ T cell responses ranged from 0.5% to 4.2% at the primary peak phase (W2–W6), then decreased to 0.3–1.1% at the contraction phase and started to re-emerge from 0.8% to 3.7% at the first re-emergence phase (W18–26). For TRN proliferative CD8^+^ T cells, maximal responses ranged from 0.9% to 4.8% at the primary peak phase (W2–W6), then decreased to 0.3–0.8% at the contraction phase and started to re-emerge from 1.1% to 2.1% first at W18–26. Maximal Env and Pol proliferative CD8^+^ T cell responses remained below 1.5% for all animals and during all phases (Table S1).

Of note, animal BX83 experienced a transient T cell activation (W3 to W8 PI) for undetermined reasons. Therefore we were not able to correctly assess the level of the primary peak of SHIV-specific proliferative CD8^+^ T cell response with this type of assay.

With regards to IL-2 responses, discrete subsets of antigen-specific IL-2 secreting CD8^+^ T cells (<0.05% of total live CD8^+^ T cells) were sporadically measured mostly within non-proliferating cells (data not shown).

During the primary phase (W3 PI), both IFN-γ ELISPOT and ICS assays on PBMCs cultured for 6 days failed to detect IFN-γ producing cells. These results suggested that the absence of IFN-γ was not due to a difference of sensitivity in the two methods but rather that the immediate IFN-γ responses measured after 18 h of culture are mainly composed of short-life effector cells that were prone to die within few days of culture (data not shown).

Altogether, these results demonstrated that the IM and ID-EP delivery of a single moderate dose of replication-limited SHIV-based DNA vaccine induced detectable, long-lasting and broad SHIV-specific CD8^+^ T cells with polyfunctional recall capacity (proliferation +, Granzyme B +), but limited immediate effector functions (IFN-γ-, IL-2-) among all vaccinated animals. These responses qualitatively resembled the responses observed in our previous studies using a single high dose of non-replicating lentiviral based-DNA vaccine.

### 3. SHIV-specific CD4^+^ T cell responses in immunized macaques

**Figure 3:**
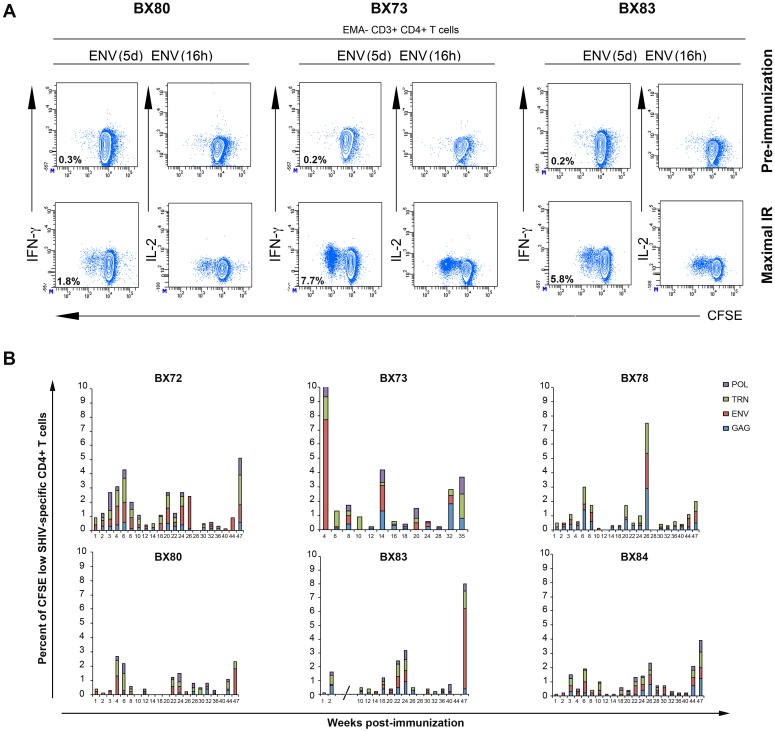
SHIV-specific CD4^+^ T cell recall responses. PBMCs were processed and labeled using the procedure described in the legend of Figure 2. **A)** Flow cytometry data analyses were performed by gating on low FSC/SSC, EMA-, CD3^+^ and CD4^+^ T cell populations (colored in blue). Representative results obtained for macaques BX73, 80 and 83 at the pre-immunization time point and at the time of maximal immune response (IR) for Env antigen are displayed. Frequencies for Env-specific responses are reported as the percent of proliferating CD4^+^ T cells after the subtraction of backgrounds obtained with cells cultured for 5 days with medium alone and restimulated for 6 h with relevant peptides pools. **B)** Summary of the frequency of proliferating (CFSE low) CD4*^+^* T cells detected against each indicated antigen (Gag (blue), Env (red), TRN (green), Pol (purple)) in each immunized animal (BX72, 73, 78, 80, 83, 84) following weeks post-immunization. Of note, results obtained between W3 and W8 for BX83 were excluded due to non-specific T cell hyperactivation (indicated by an interrupted x-axis).

In our polyfunctional recall assay we included cell surface staining for CD4^+^ T cells to examine vaccine-specific CD4^+^ T cell responses. Examples of data obtained with the pre-immune samples and those showing maximal proliferative responses against Env antigens with samples from 3 animals (BX80, 73, 83) are shown in Figure 3A. Similar to vaccine-specific CD8^+^ T cell responses, CD4^+^ T cells showed sustained proliferative ability (>3 divisions) but no immediate effector functions (IFN-γ-, IL-2-) in the 6 days culture assay. Interestingly, similar to the data obtained in our previous lentiviral-based DNA vaccine study, the vaccine-specific CD4^+^ T cell responses progressed through the same dynamic as the one observed with CD8^+^ T cell responses. Indeed, primary peaks of expansion were observed between W2 and W6 PI, followed by a contraction phase between W8 and W20 PI, and then late re-emergence peaks (W22 to W47 PI), in the absence of booster immunization, as summarized in Figure 3B. Vaccine-specific CD4^+^ T cells were directed against all tested antigens. The maximal proportion of total antigen responses measured across the longitudinal analysis in all animals remained below 5% of total CD4^+^ T cells, except for 3 animals (BX73, 78 and 83) that reached levels above 7%, mostly due to a high Env response, at single distinct time-points (W4 for BX73, W26 for BX78 and W47 for BX83). Maximal responses measured in all animals at various time points PI ranged from 0.3% to 2.9% and from 0.8% to 2.2% of total live CD4^+^ T cells for Gag and TRN respectively. For Pol and Env, these responses varied from 0.3% to 1.6% and from 0.3% to 7.7% respectively (Table S2). Overall, these results indicate that in parallel to vaccine-specific CD8^+^ T cell responses, broad and long-lasting vaccine specific CD4^+^ T cell responses have been induced in all vaccinated animals, and may contribute to the longevity of vaccine-specific T and B cell responses.

### 4. Polyfunctional analysis of vaccine-specific memory T cells expanded under antigenic and homeostatic driven proliferation conditions

**Figure 4:**
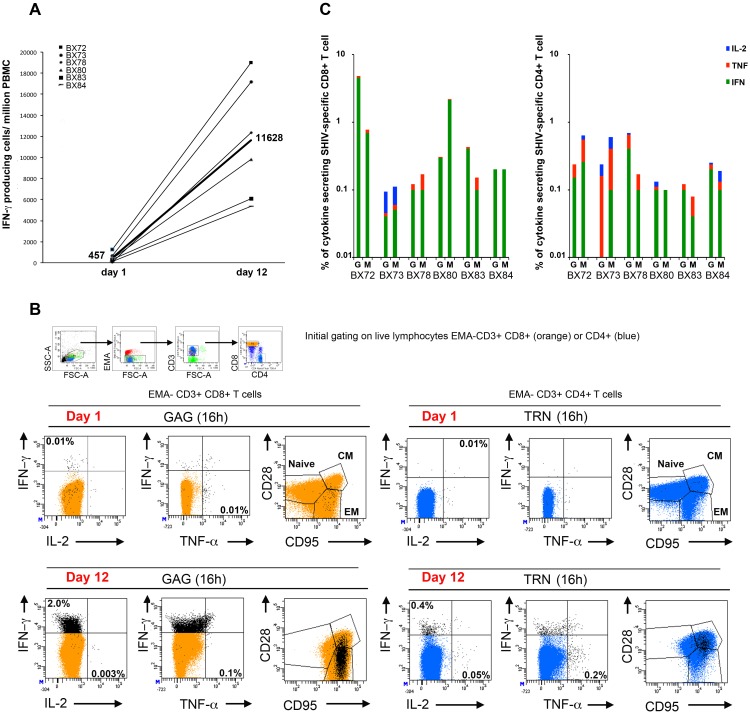
Vaccine-specific PHPC T cell responses at late time points post-immunization. **A)** PBMCs were isolated from blood samples taken between W40 and W47 PI for all animals (W28 to 35 PI for BX73). A portion of cells from each animal was used for ELISPOT assay to detect IFN-γ producing cells in response to medium, Gag and TRN pools of peptides (day 1). The other portion was cultured for 11 days in the presence or absence of relevant peptide pools, in medium supplemented on day 3 with recombinant simian IL-2 only and on day 7 with recombinant simian IL-2, IL-15 and IL-7 cytokines. On day 11, cells were collected, counted and used for ELISPOT assay using the same conditions as day 1 ELISPOT, to detect IFN-γ producing cells in response to Gag and TRN peptides (day 12). This assay is referred as PHPC assay for memory precursors with high proliferative capacity. Numbers of IFN-γ producing cells obtained per million of PBMCs against Gag + TRN at day 1 and day 12 are indicated in the *y*-axis. The number of median responses obtained among all 6 animals are represented (n = 457 spots/10^6^ at day 1 and n = 11628 spots/10^6^ at day 12). **B)** Memory phenotyping of Gag and TRN-specific T cells for animal BX72 at W40 PI. Day 1 and day 11 cultured PBMCs, as described in section A, were restimulated for 16 h in the presence or absence of Gag or TRN pool of peptides in medium containing Brefeldin A. Cells were then surface-stained with EMA, CD3, CD8, CD4, CD28, CD95 mAbs, permeabilized and stained with IFN-γ, IL-2 and TNF-α mAbs. Cells were gated on live lymphocytes (low FSC/SSC, EMA-, CD3^+^), bright CD8^+^ T cell populations (orange color) and CD4^+^ T cell populations (blue color). Antigen-specific T cells were identified by their capacity to secrete one or more cytokines (black dots). For simplicity, the percentage of total IFN-γ+ or TNF-α+ or IL-2+ antigen specific responses (black dots) obtained are indicated after the subtraction of background obtained with cells cultured with medium only. All black dots were superimposed to the total CD8^+^ or CD4^+^ T cell population and plotted against EM and CM memory markers (CD28 and CD95). **C)** The same procedure as described in B was performed on day 11-cultured PBMCs that have been isolated at W47 PI (W35 PI for BX73) from all six animals. The histograms represent the percentage of total IFN-γ+ (green) or TNF-α+ (red) or IL-2+ (blue) antigen-specific T cell responses obtained against Gag and TRN (indicated by G and M respectively in the histogram) within CD8^+^ (left histogram) and CD4^+^ (right histogram) T cells. Individual dot plot analysis for cytokine and memory phenotype shown in the Figure 4C, are displayed in supplemental Figure S3 and S4.

The difference in IFN-γ responses detected by the 18 h ELISPOT assay and the 6-day polyfunctional recall assay, clearly indicated that these two types of assays identify different subsets of antigen-specific T cells. While the IFN-γ ELISPOT assay is thought to identify effector T cells with low proliferative capacity, another peptide-based cultured IFN-γ ELISPOT assay has been used to quantify antigen-specific memory T cells precursors with high proliferative capacity (PHPC assay) [38]. This assay is thought to assess central memory T cells that require more time to expand and give rise to cells with immediate effector functions. Importantly, in untreated HIV seropositive individuals, strong PHPC responses correlated with low viremia and high CD4^+^ counts [42]. The memory phenotype analysis performed on day 1 stimulated PBMCs (Figure 1C) clearly indicated the presence of antigen-specific central memory cells within the cytokine secreting population. Antigen-specific CM T cells were also found within the population of cells that proliferate only in our 6 day culture assay (data not shown). We developed and performed a slightly modified PHPC assay to examine peripheral blood samples of vaccinated animals collected at W40 to W47 PI time points (W28 to W35 PI for BX73). Briefly, PBMCs were expanded for 11 days in culture supplemented or not with antigen on day 1 followed with recombinant simian IL-2 only (to force short life effectors to die) on day 3 and then with a cocktail of recombinant simian IL-2, IL-15 and IL-7 cytokines on day 7 (to promote expansion of memory CD8^+^ and CD4^+^ T cells). On day 11, cells were collected, counted and tested in the IFN-γ ELISPOT assay and polychromatic flow cytometry assay. The results shown in Figure 4A clearly illustrated that the number of SHIV-specific IFN-γ immediate effector cells could be greatly expanded by culturing the cells for 11 days in presence of antigenic and homeostatic signals. Among all six vaccinated animals, we measured and calculated a mean of Gag+TRN response of 457 spots/10^6^ PBMCs (range 57–1248, n = 6) for the day 1 ELISPOT assay and a mean of Gag+TRN response of 11628 spots/10^6^ PBMCs (range 5387–19007, n = 6) for the day 12 ELISPOT assay. This represented an average 60 fold increase of expansion. Each of the six macaques also had positive PHPC responses against Gag GW9 and/or Nef RM9 antigen (Figure S2B). We next assessed the phenotype and function of the Gag and TRN-specific T cells at day 1 and day 12 similar to the experiment depicted in Figure 1C. PBMCs, fresh or cultured for 11 days, were stimulated for 16 h with Gag or TRN pools of peptides or with medium only, in presence of Brefeldin A. Cells were then surface-stained with anti-CD3, CD4, CD8, CD28 and CD95 mAbs in the presence of EMA (for dead cell exclusion). After a permeabilization step, intracellular staining was performed with anti-IFN-γ, TNF-α and IL-2 mAbs. A representative example is illustrated in Figure 4B for animal BX72 on day 1 and day 12 starting with PBMCs collected at W40 PI. For the day 1 assay, this analysis identified Gag specific-CD8^+^ T cells capable of secreting IFN-γ and/or TNF-α as well as TRN-specific CD4^+^ T cells capable of secreting IFN-γ and/or IL-2 with a proportion of single or multiple cytokine secreting cells around 0.01% of total live CD8^+^ or CD4^+^ T cells. The vast majority of vaccine-induced T cells expressed EM markers for CD8^+^ T cells and CM markers for CD4^+^ T cells. At day 12, Gag and TRN cytokine secreting-specific T cells had greatly expanded. Gag specific-CD8^+^ T cells capable of secreting mostly IFN-γ (2.0%) and TNF-α (0.1%) were found as well as TRN-specific CD4^+^ T cells capable of secreting IFN-γ (0.4%), IL-2 (0.05%) and TNF-α (0.2%). When plotted against memory markers, expanded Gag-specific CD8^+^ T cells were distributed within EM and CM T cell populations, while expanded TRN-specific CD4^+^ T cells mostly remained within the CM T cell population. These results indicate that within the CD8^+^ T cell compartment, CM specific T cells have expanded to maintain and enrich their initial pool size as well as to promote the massive development of secondary immediate effector EM type of cells.

We next repeated the polyfunctional flow cytometry analysis on PBMCs that had been expanded for 12 days from samples from all six animals collected at the latest time point (W47 or W35 PI). A representative profile of cytokines found in expanded Gag and TRN (indicated by M in this figure) specific CD8^+^ and CD4^+^ T cells is illustrated in Figure 4B. Most expanded antigen-specific CD8^+^ and CD4^+^ T cells expressed mainly IFN-γ with a range of responses between 0.1 and 1% (median = 0.4%, n = 24) within total live CD8^+^ or CD4^+^ T cells. Samples from two animals mounted stronger IFN-γ responses reaching 4.5% for Gag (BX72) and 2.2% for TRN (BX80) specific CD8^+^ T cells. We also detected IL-2, albeit at levels lower than IFN-γ, (median = 0.02%, n = 24) and TNF-α (median = 0.06%, n = 24) cytokine secreting Gag and TRN-specific T cells, indicating that expanded cells could be heterogeneous functional cells. The expanded Gag and TRN-specific CD8^+^ T cells were within EM and CM T cell populations. Gag and TRN-specific CD4^+^ T cells appeared mostly within the CM T cell population (individual dot plot analysis for cytokine and memory phenotype used in the Figure 4C are displayed in Figures S3 and S4).

Altogether, these results demonstrated that, at late time points PI with CAL-SHIV-IN^−^ DNA, antigen-specific CM and EM T cells appeared expanded in blood and upon extended culture in the presence of appropriate antigenic and homeostatic signals these cells acquired effector functions. Thus, a single immunization with our DNA vaccine allowed the generation and persistence of vaccine-specific memory T cells with immediate as well as recall effector functions in blood of vaccinated macaques. In addition, polychromatic flow cytometry analysis also clearly identified expanded IFN-γ effector cells measured in PHPC assay by ELISPOT as antigen-specific EM and CM type of T cells. This confirmed that the evaluation of T cell responses by PHPC assay might be an attractive tool for testing the functionality of prophylactic vaccines.

## Discussion

In this study, we evaluated the immunogenicity of a novel non-integrative one-cycle SHIV-based DNA vaccine under the control of a constitutively active LTR in macaques after a single DNA immunization. Unlike other reports, the longitudinal phenotypic and functional analysis of vaccine-induced T cell responses was repeated at relatively short intervals across almost a year PI to precisely monitor the dynamics of the responses. The combined single-cycle vaccine and delivery strategies (IM+ID-EP) promoted the development and maintenance of vaccine-specific CM and EM CD8^+^ and CD4^+^ T cells with immediate as well as readily inducible effector functions.

Interestingly, our ongoing evaluation of humoral responses revealed antibody responses to Gag antigens in serum samples from all vaccinated macaques and to HIV Env in half the animals. In our hands, this is the first DNA vector/strategy that induced both potent T cell and antibody responses compared to our former HIV DNA vaccine prototypes that induced potent T cell responses but failed to induce any detectable humoral response both in mouse and NHP models [30], [31]. Of interest though was the differing kinetics of antibody titers to the 2 antigens. While the peak of Gag antibody responses appeared early PI, Env antibody responses augmented markedly later suggesting differential responses and sustained antigen presentation of Env proteins.

Integration competent or non-integrative lentiviral vectors for SIV/HIV vaccines have been shown to achieve sustained expression of viral antigens and promote differing level of protective immune responses without pathologic outcome [43]. However, the protection afforded by these constructs leaves ample room for improvement. The CAL-SHIV-IN*^−^* DNA construct is novel, combining lentiviral sequences from 3 different species: simian (*gag*, *pol* (deleted of *integrase* sequence), *nef*, *vif*, *vpx*, *vpr*), human (*env*, *tat*, *rev*, *vpu*) and caprine (5′ and 3′ LTRs). In addition, the removal of SIV integrase (IN) conferred a high degree of safety, because not only INs are LTR sequence specific but total absence of IN abolishes the retrovirus-specific integration of the vaccine genome resulting in a non-integrative, single-cycle lentivector. Unlike primate lentiviruses, the CAEV LTR promoter is Tat-independent allowing for constitutive expression of viral proteins in various cell lineages. Finally, the simultaneous delivery of vaccine DNA into muscle and dermis was meant to promote DNA uptake by myocytes, keratinocytes and recruited and resident antigen presenting cells (APC) such as macrophages and dendritic cells, especially epidermal Langerhans cells augmented by the electroporation procedure, as previously described [37], [44]. From the episomal plasmid DNA, viral RNA and proteins would be produced and assembled into viral particles capable of infecting all HIV target cells including CD4^+^ T lymphocytes, macrophages and dendritic cells. However, viral replication would be limited only to one cycle of infection and viral proteins would be transiently expressed by non-integrated circular viral DNA without production of new infectious particles. Altogether, we hypothesized that the design of our novel lentivector could allow for an amplification and diversification of antigen presentation *in vivo* but in a self-limited manner and under balanced inflammatory conditions that should promote optimal immune responses.

Indeed, based on ELISPOT responses, a substantial increase was noted in the vaccine-specific IFN-γ immediate responses in our current study relative to the minimal responses (<150 SFCs) observed in our previous studies that used repeated low dose or single high dose of the non-integrative non-replicative HIV DNA vaccine, Δ4-SHIV-_KU2_ [30], [31]. Thus, the cumulative mean responses against Gag, Tat, Rev and Nef antigens, across one year follow up PI, reached an average of 726+/− 430 SFCs/million (range = 225–1140, n = 6 animals). Interestingly, in another study, cynomolgus macaques primed once and boosted twice with a DNA vaccine vector administered ID with EP similar to our protocol, developed substantial levels of IFN-γ ELISPOT responses against Gag, Tat, Rev and Nef antigens (around 1000 SFCs/million) which remained detectable almost 3 years after the last immunization [37]. These results suggest that the observed improvement in immediate effector cell responses may result from both the delivery process (ID-EP) and the single-cycle associated with our current construct. Accordingly, during a preliminary experiment, we immunized two groups of humanized SCID mice with CAL-SHIV-IN^−^ by IM or ID-EP routes and found augmented IFN-γ ELISPOT responses in the both groups. These results support the notion that SIV and HIV DNA vaccines, can be highly immunogenic *in vivo* when delivered in combination with electroporation [45] or associated with single-cycle replication [46].

A closer comparison with other existing platforms like SIV DNA electroporation adjuvanted with specific cytokines or chemokines remains difficult to perform because of the systematic use of prime-boost strategy that generate higher antigen-specific IFN-γ responses than ours, but mostly after antigen boost [23], [24], [46]. Interestingly, the intradermal delivery of a single dose of Dermavir which consist in a formulation aimed at targeting Langerhans cells and containing a plasmid DNA able to express HIV viral proteins that assemble as non-infectious particles, promoted the development of vaccine-specific persisting IFN-γ ELISPOT responses (48 weeks PI) as well as PHPC responses in vaccinated HIV infected patients under antiretroviral treatment [38], [47]. Similarly, in our present study, using the ID-EP procedure and SHIV-based DNA vaccine we promoted a profile of vaccine-specific T cell responses with persisting IFN-γ ELISPOT as well as PHPC responses in all vaccinated animals. Thus, these results indicate that skin targeted vaccine delivery [44] might be an important vaccination strategy for induction of PHPC type of responses. Recently, PHPC responses were also largely observed in HIV Elite controllers and found to be associated with central memory responses [42], [48]. In our study, using memory markers (CD28 and CD95) commonly used in macaque studies [48] the PHPC protocol demonstrated that vaccine specific T cells strongly expanded in response to antigen, IL-15 and IL-7 and mostly regenerated CM (CD28+ CD95+) and EM (CD28− CD95+) type of T cells. A recent study has also demonstrated the existence of stem cell memory T cells (Tscm) which express CD28 and CD95 markers like CM T cells but also additional markers associated with naïve like phenotype (CD45RA, CCR7, CD127, CD27). These cells are generated during the acute phase of infection by SIVmac239 in macaques and preferentially survive for a long time following the elimination of antigen due to their unique homeostatic properties [49]. Whether our strategy of vaccination induced such antigen-specific Tscm that may potentially contribute to the longevity of the immunity in the absence of booster immunization remains to be determined.

Although the longevity and modulation of immune responses was already observed in our previous Δ4-SHIV-_KU2_ vaccine studies [30], [31], the mechanisms underlying the observed dynamics of the immune responses following this single immunization remain to be elucidated. By performing a longitudinal characterization of immune responses following a single DNA immunization and using the same immunogenicity read-out assay (polyfunctional assay depicted in Figure 2 and 3) through independent studies in different animal models (mouse and rhesus and cynomolgus macaques), we consistently observed an initial expansion, a contraction and late reemergence phases of vaccine-specific CD8^+^ and CD4^+^ T cells in absence of additional immunization or antigen boost. These reemergence phases occurred both with our non-integrative non-replicative vaccine (Δ4-SHIV-_KU2_) as well as our novel non-integrative single-cycle vaccine (CAL-SHIV-IN^−^), two non-persisting vectors. Although evidence of DNA persistence was clearly negative in our previous murine study, it seems likely that episomal DNA and/or DNA transduced cells may survive for extended periods of time e.g. in draining lymph nodes or other sites of the immunized animals, leading to reactivation of viral protein production once such responses have dipped below a specific inhibitory threshold. The recall of SHIV memory responses and/or the stimulation of novel antigen-specific T cells as well as the dynamic of antibody responses certainly suggest reexpression of antigen. However, sensitive RT-PCR analysis failed to detect viral RNA in the plasma of the vaccinated animals (data not shown). It is possible too that the multisite delivery of the vaccine (IM+skin/electroporation) may result in various cell lineage transduction with different kinetics of protein production from the vector, though our previous study with Δ4-SHIV-KU2 showed a similar pattern even though the vaccine was administered IM only. Another potential explanation for the longevity and resurgence of immune responses may be due to homeostatic mechanisms. In a previous study, the introduction of IL-15 plasmid DNA in one macaque 40 weeks post-immunization with a single dose DNA immunization allowed the reemergence of circulating Ag-specific memory T cells with high proliferative capacity in the periphery [31]. Ongoing studies are planned to investigate this mechanism in detail. To address this hypothesis, we will examine the potential longevity of vaccine transduced cells in different anatomic sites of immunized animals, including muscle, skin (sites of injection), the digestive and reproductive tract and various lymphoid tissues. We will first screen these tissues with our ultra sensitive (nested) PCR, with primers specific to CAEV LTRs to detect any residual vaccine DNA in both mononuclear and tissue cells. Samples that are found positive will then be examined by immunohistochemistry using anti-Gag p27 specific antibodies and/or In Situ Hybridization to detect viral RNA to confirm expression of this residual DNA. However, in the context of the current experiment, we could not predict the timing (or the occurrence) of the resurgence of immune responses (similarly observed in mice immunized with the same vector), and such tissue sampling is invasive and somewhat risky during an immunogenicity phase and thus, such collections were not performed.

Finally, the identification of persistent responses toward two specific Gag (GW9) and Nef (RM9) antigens that are found early during fully replicative SIVmac239 infection and rapidly lost by mutation escape [41] argue that our single-cycle limited CAL-SHIV-IN^−^ had recapitulated the initial acute phase of infection only. In the mouse model of LCMV infection, the maintenance of CD8^+^ memory T cells generated during acute infection is independent of Ag [50], in contrast to the maintenance of CD8^+^ T cell memory during chronic infection that is dependent on persistent Ag stimulation. In mathematical modeling of a primary CD8^+^ T cell response to an acute infection, the homeostasis of the memory T cell pool without antigen stimulation has been associated with a Tm proliferation capable of balancing the number of cells that enter the pool against the number that leave the pool to maintain a stable pool of memory T cells in the long run [51], [52]. We can speculate that after an extensive absence of antigen, vaccine-specific memory T cells, mainly located in lymphoid tissues, alter their trafficking property by regulating the expression of CCR5 (migration to extra lymphoid effector sites) and CCR7 (homing marker for lymphoid tissues) markers, and this may allow the cells to reach the periphery and participate to the reemergence phases observed in our study. Whether the dynamic of vaccine-specific T cell responses we observed in the peripheral blood, is an intrinsic property of our lentiviral-based vectors or rather linked to the single immunization strategy remains to be addressed. Our study also emphasized the importance to perform longitudinal characterization of immune responses rather than few selected time points to better harness the dynamics of the vaccine-T cell responses and determine at which stage these responses can be modulated by the Ag or homeostatic cytokines boosts. Of importance, such characterization should be also extended to other anatomic locations since immune T cell responses in lymph nodes have been reported to better correlate with protection against pathogenic viral challenge in LAV-infected macaques [17].

In conclusion, our study demonstrated that a single administration, by IM+ID-EP, of a self-limited replicative SHIV-based DNA vaccine allowed efficient generation and maintenance of a combination of memory T cell responses with immediate and readily inducible effector functions (PHPC responses) in the absence of ongoing antigen stimulation. Whether such combined strategies can be as efficient as vectors allowing persisting antigen expression in reducing SIV viral replication after challenge in vaccinated animals needs further exploration.

## Supporting Information

Figure S1 **Schematic representation of CAL-SHIV-IN^−^.** CAL-SHIV-IN*^−^* is a derivative of the pathogenic SHIV-KU2 [12] whose SIV LTRs have been removed and replaced by CAEV LTRs and the SIV *integrase* coding sequences were deleted. SIV genes and HIV genes in our chimeric construct are indicated by different color.
(TIF)

Figure S2 **A) MHC haplotypes.** The MHC haplotype (H1 to H6) for each animal was determined by microsatellite analysis as described elsewhere [35], [36]. The MHC map is indicated on top of microsatellite markers. For each chromosome, intact and recombinant haplotype are color-coded (H4 dark pink, H1 blue, H2 light pink, H3 light orange, H6 green). $ = Probable allelic mutation. Rec (Hx-Hy) = Recombinant haplotype between Hx and Hy. **B) IFN-γ** **responses to common Gag and Nef MHC-class I restricted epitopes.** PBMCs isolated from blood samples taken at W47 post-immunization (*W35 PI for BX73) were used for ELISPOT assay to detect IFN-γ producing cells in response to Gag or Nef pools of peptides as well as individual peptide named Gag GW9 (GPRKPIKCW) and Nef RM9 (RPKVPLRTM). Cells were cultured in presence or absence of peptides for 18 h (W47 d1). In addition, cells were cultured for 11 days in presence or absence of Gag or TRN pools of peptides supplemented on day 3 with mamu IL-2 and on day 7 with a cocktail of IL-2 (10 U/ml), IL-15 (10 U/ml) and IL-7 (500 ng/ml). Expanded cells were then used for IFN-γ ELISPOT assay and cultured in presence or absence of indicated peptides for 18 h (W47 d12). Numbers of IFN-γ producing cells obtained per million of PBMCs against tested antigens are indicated in the *y*-axis.
(TIF)

Figure S3 **Dot plot analysis of** **Figure 3B** **for animals BX 72, 73 and 78.**
(TIF)

Figure S4 **Dot plot analysis of** **Figure 3B** **for animals BX 80, 83 and 84.**
(TIF)

Table S1 **Maximal proliferative SHIV-specific CD8^+^ T cell responses detected during the indicated phases for each animal in response to the indicated antigens.** Calculated total Ag responses are also reported.
(TIFF)

Table S2 **Maximal proliferative SHIV-specific CD4^+^ T cell responses detected during the indicated phases for each animal in response to indicated antigen.** Calculated total Ag responses are also reported.
(TIFF)
