# Supplementary figures and images for: Long-Term Central and Effector SHIV-Specific Memory T Cell Responses Elicited after a Single Immunization with a Novel Lentivector DNA Vaccine
Source: PLoS One. 2014 Oct 22;9(10):e110883. doi: 10.1371/journal.pone.0110883 (PMC4206452; doi:10.1371/journal.pone.0110883)

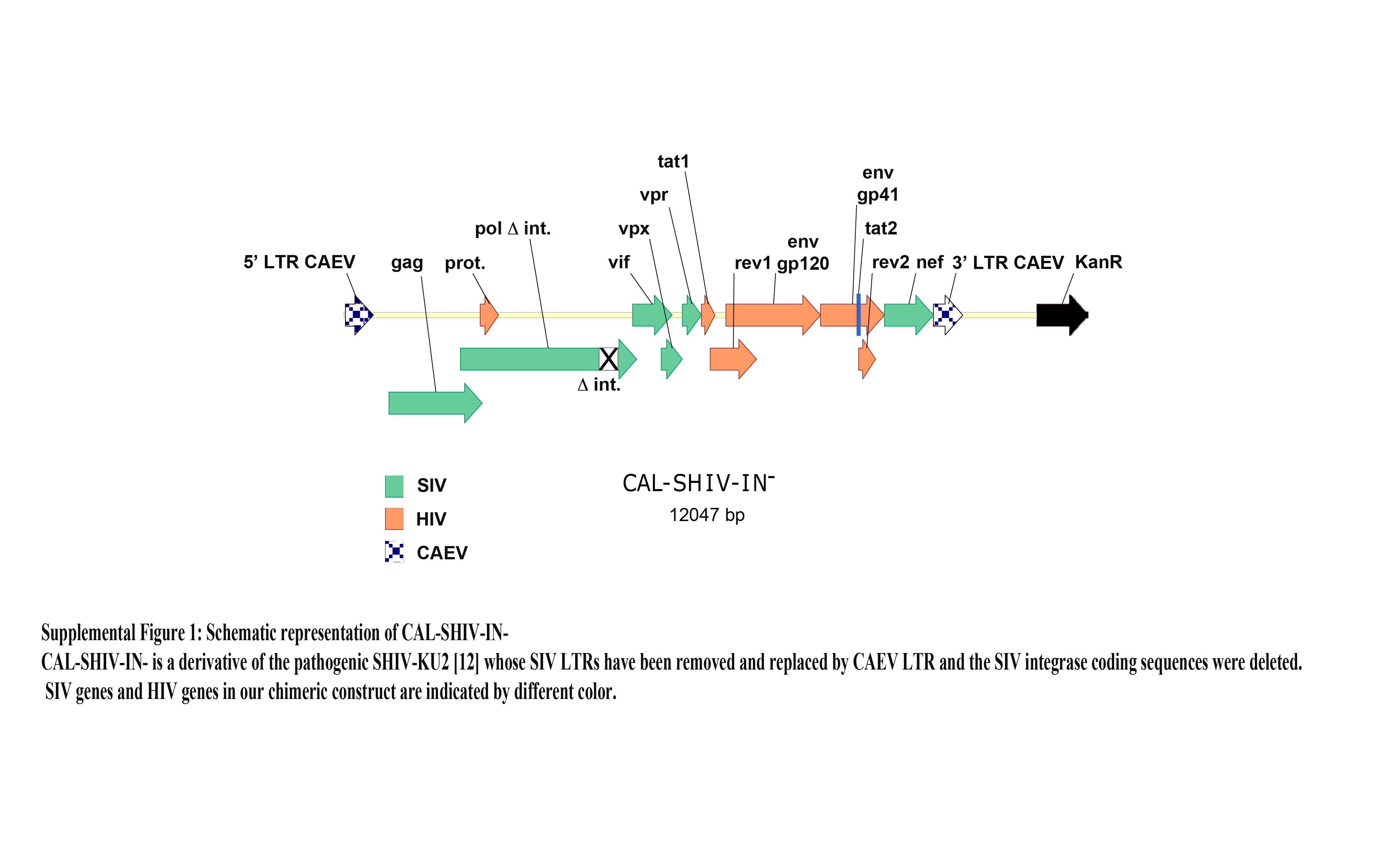

Supplement: Figure S1 — Schematic representation of CAL-SHIV-IN−. CAL-SHIV-IN− is a derivative of the pathogenic SHIV-KU2 [12] whose SIV LTRs have been removed and replaced by CAEV LTRs and the SIV integrase coding sequences were deleted. SIV genes and HIV genes in our chimeric construct are indicated by different color. (TIF) [file pone.0110883.s001.tif]

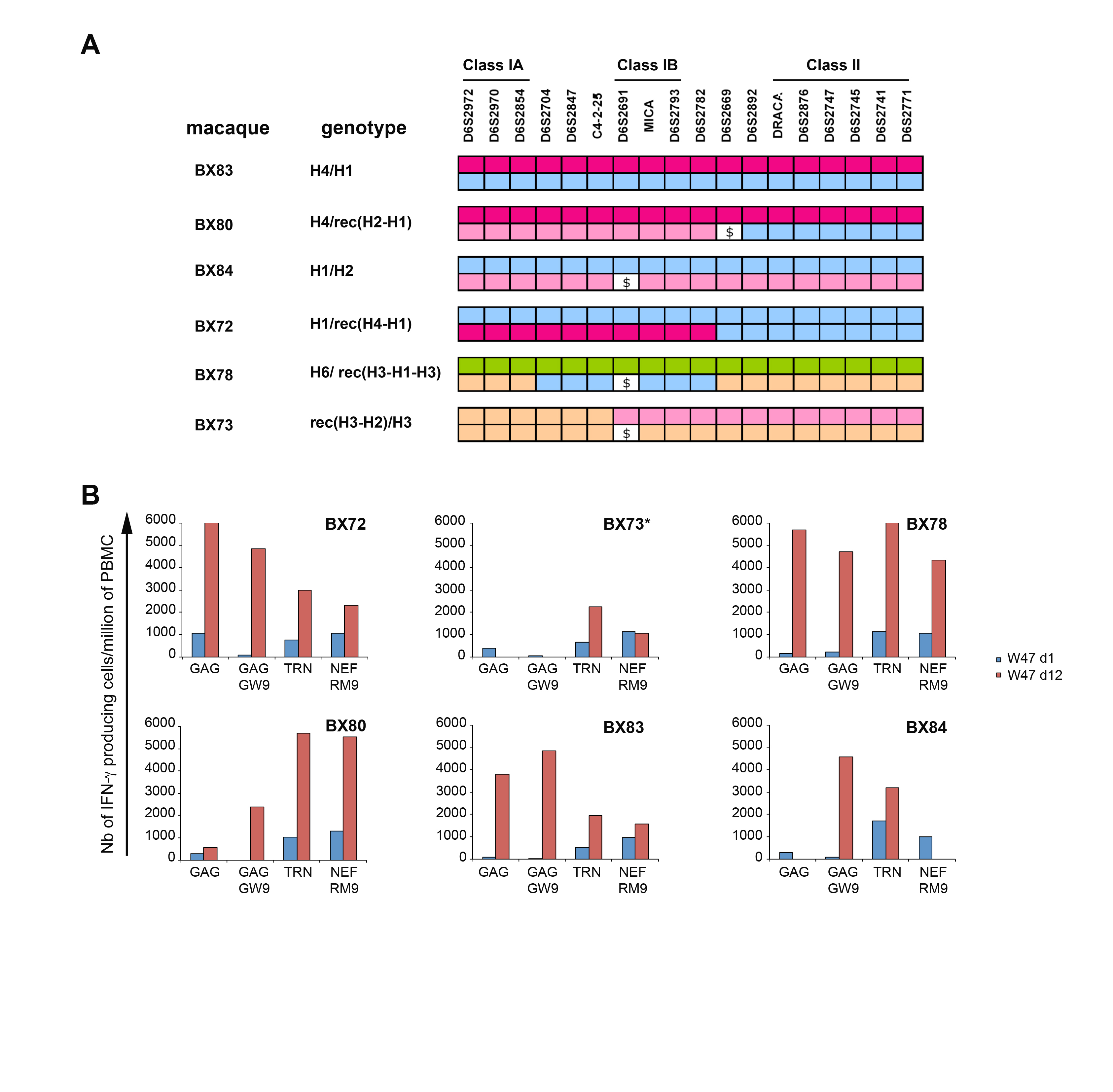

Supplement: Figure S2 — A) MHC haplotypes. The MHC haplotype (H1 to H6) for each animal was determined by microsatellite analysis as described elsewhere [35], [36]. The MHC map is indicated on top of microsatellite markers. For each chromosome, intact and recombinant haplotype are color-coded (H4 dark pink, H1 blue, H2 light pink, H3 light orange, H6 green). $ = Probable allelic mutation. Rec (Hx-Hy) = Recombinant haplotype between Hx and Hy. B) IFN-γ responses to common Gag and Nef MHC-class I restricted epitopes. PBMCs isolated from blood samples taken at W47 post-immunization (*W35 PI for BX73) were used for ELISPOT assay to detect IFN-γ producing cells in response to Gag or Nef pools of peptides as well as individual peptide named Gag GW9 (GPRKPIKCW) and Nef RM9 (RPKVPLRTM). Cells were cultured in presence or absence of peptides for 18 h (W47 d1). In addition, cells were cultured for 11 days in presence or absence of Gag or TRN pools of peptides supplemented on day 3 with mamu IL-2 and on day 7 with a cocktail of IL-2 (10 U/ml), IL-15 (10 U/ml) and IL-7 (500 ng/ml). Expanded cells were then used for IFN-γ ELISPOT assay and cultured in presence or absence of indicated peptides for 18 h (W47 d12). Numbers of IFN-γ producing cells obtained per million of PBMCs against tested antigens are indicated in the y-axis. (TIF) [file pone.0110883.s002.tif]

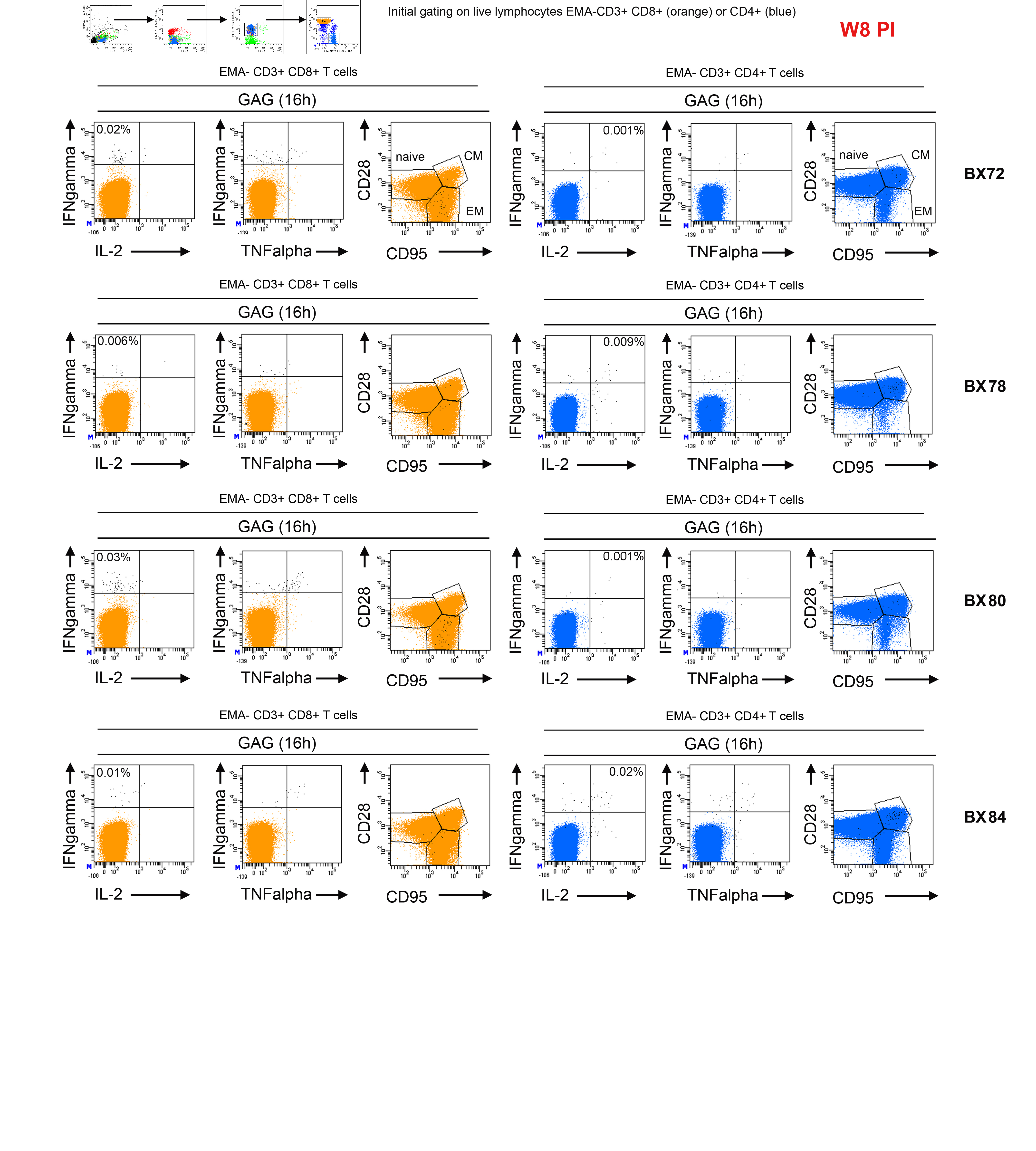

Supplement: Figure S3 — Dot plot analysis of Figure 3B for animals BX 72, 73 and 78. (TIF) [file pone.0110883.s003.tif]

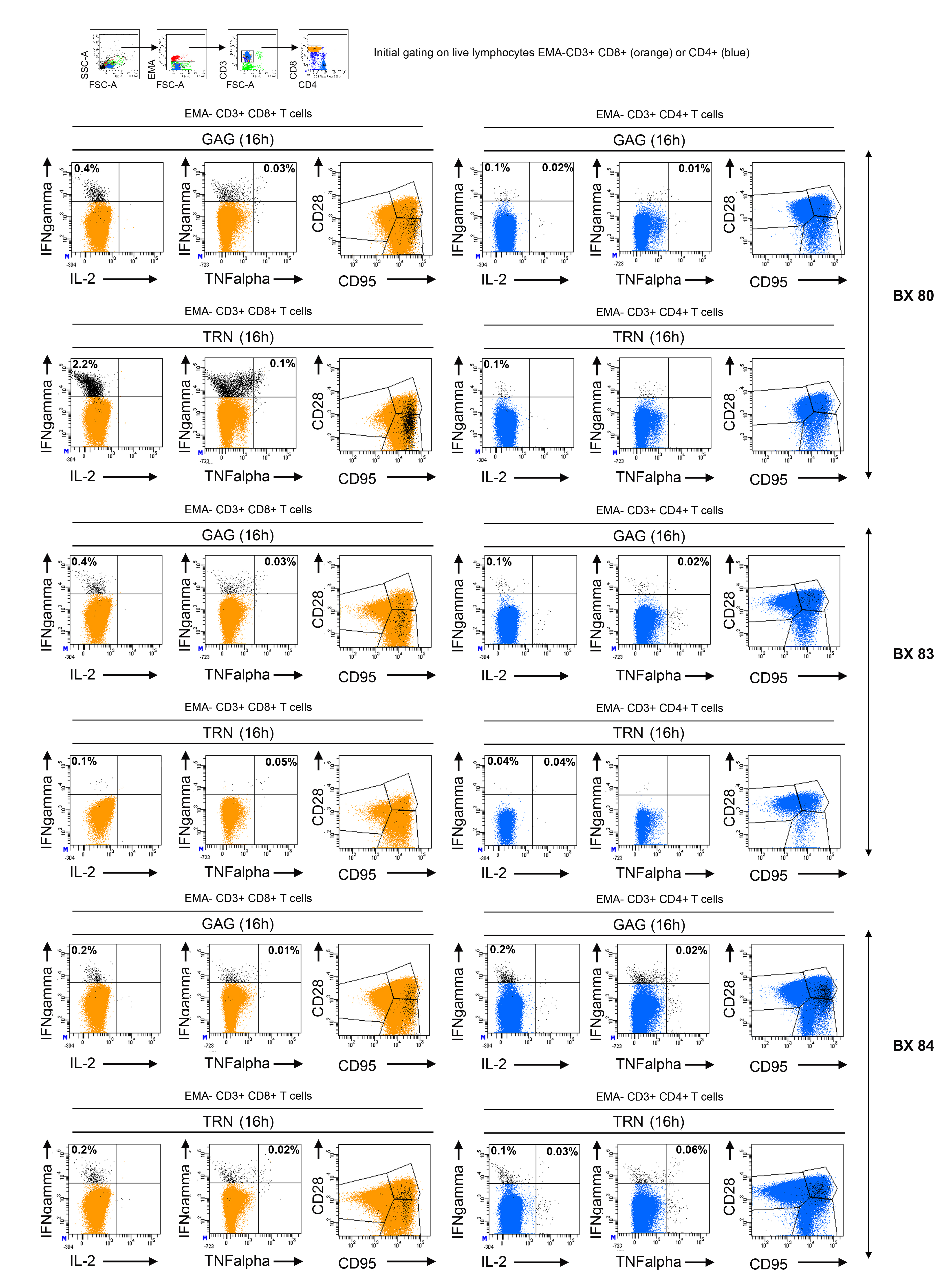

Supplement: Figure S4 — Dot plot analysis of Figure 3B for animals BX 80, 83 and 84. (TIF) [file pone.0110883.s004.tif]
